# Severe Obesity in Young Women and Reproductive Health: The Danish National Birth Cohort

**DOI:** 10.1371/journal.pone.0008444

**Published:** 2009-12-24

**Authors:** Ellen A. Nohr, Nicholas J. Timpson, Camilla S. Andersen, George Davey Smith, Jørn Olsen, Thorkild I. A. Sørensen

**Affiliations:** 1 Department of Epidemiology, University of Aarhus, Aarhus, Denmark; 2 Department of Social Medicine, University of Bristol, Bristol, United Kingdom; 3 The Institute of Preventive Medicine, Copenhagen University Hospital, Copenhagen, Denmark; 4 Department of Epidemiology, School of Public Health, University of California Los Angeles, Los Angeles, California, United States of America; The University of Adelaide, Australia

## Abstract

**Background:**

Little is known about reproductive health in severely obese women. In this study, we present associations between different levels of severe obesity and a wide range of health outcomes in the mother and child.

**Methods:**

From the Danish National Birth Cohort, we obtained self-reported information about prepregnant body mass index (BMI) for 2451 severely obese women and 2450 randomly selected women from the remaining cohort who served as a comparison group. Information about maternal and infant outcomes was also self-reported or came from registers. Logistic regression was used to estimate the association between different levels of severe obesity and reproductive outcomes.

**Principal Findings:**

Subfecundity was more frequent in severely obese women, and during pregnancy, they had an excess risk of urinary tract infections, gestational diabetes, preeclampsia and other hypertensive disorders which increased with severity of obesity. They tended to have a higher risk of both pre- and post-term birth, and risk of cesarean and instrumental deliveries increased across obesity categories. After birth, severely obese women more often failed to initiate or sustain breastfeeding. Risk of weight retention 1.5 years after birth was similar to that of other women, but after adjustment for gestational weight gain, the risk was increased, especially in women in the lowest obesity category. In infants, increasing maternal obesity was associated with decreased risk of a low birth weight and increased risk of a high birth weight. Estimates for ponderal index showed the same pattern indicating an increasing risk of neonatal fatness with severity of obesity. Infant obesity measured one year after birth was also increased in children of severely obese mothers.

**Conclusion:**

Severe obesity is correlated with a substantial disease burden in reproductive health. Although the causal mechanisms remain elusive, these findings are useful for making predictions and planning health care at the individual level.

## Introduction

As a consequence of the obesity epidemic, the proportion of severely obese women of childbearing age has increased considerably, which prompts research in the consequences for reproductive health of these women.

A large body of data already links prepregnancy obesity with a number of fetal and maternal complications, including subfertility, preeclampsia, gestational diabetes, fetal death, macrosomia and complicated deliveries [1]–[4]. After birth, obese women are less likely to succeed breastfeeding [5], they may have higher postpartum weight retention than other women [6], and in children born to obese mothers, a higher risk of obesity during infancy and childhood is well-documented [7]–[9].

Although the obese phenotype covers a wide range of abnormalities depending on the amount, distribution and causes of accumulated fat, most studies have defined obesity as a prepregnant body mass index (BMI = weight[kg]/height[m]^2^)≥30 and analyzed the entire group of obese individuals together. Given the approximated normal distribution of BMI, results from such studies are mainly based on obese women in the lower end of the obesity range, and knowledge about the impact of severe obesity on reproductive health is sparse. Also, when all obese women are analyzed as one group, the opportunity to study differences in risk across levels of obesity is lost. Because of the heterogeneity across the range of BMI values in the upper tail it is of importance to get a more detailed description of the interplay between severity of obesity and reproductive function. Here we compare data on a large contemporary sample of very obese women and a selected group of other women in the same population in order to study the associations between different levels of obesity and a wide range of outcomes in the mother and infant.

## Material and Methods

### The Danish National Birth Cohort

The present study was based on The Danish National Birth Cohort (DNBC). From 1996–2002, 91,387 women with a total of 100,419 pregnancies were recruited to the DNBC in early pregnancy by their GP, and approximately 60% of those invited chose to participate. Detailed descriptions of the study methods and the recruitment were published elsewhere [10]–[12]. Briefly, the main data collection consisted of two telephone interviews during pregnancy at ≈16 and ≈30 weeks of gestation and two postnatal telephone interviews when the child was ≈6 and 18 months old. Also, the woman provided two blood samples during pregnancy and a blood sample of the child taken from the umbilical cord at birth. When entering the DNBC, all women provided written informed consent that their data and biological material could be used in scientific studies of health in women and children. The main cohort study was approved by all the regional scientific ethics committees in Denmark, by the central scientific ethics committee for whole Denmark and by the Danish Data Protection Board.

### Sampling Strategy

**Figure 1:**
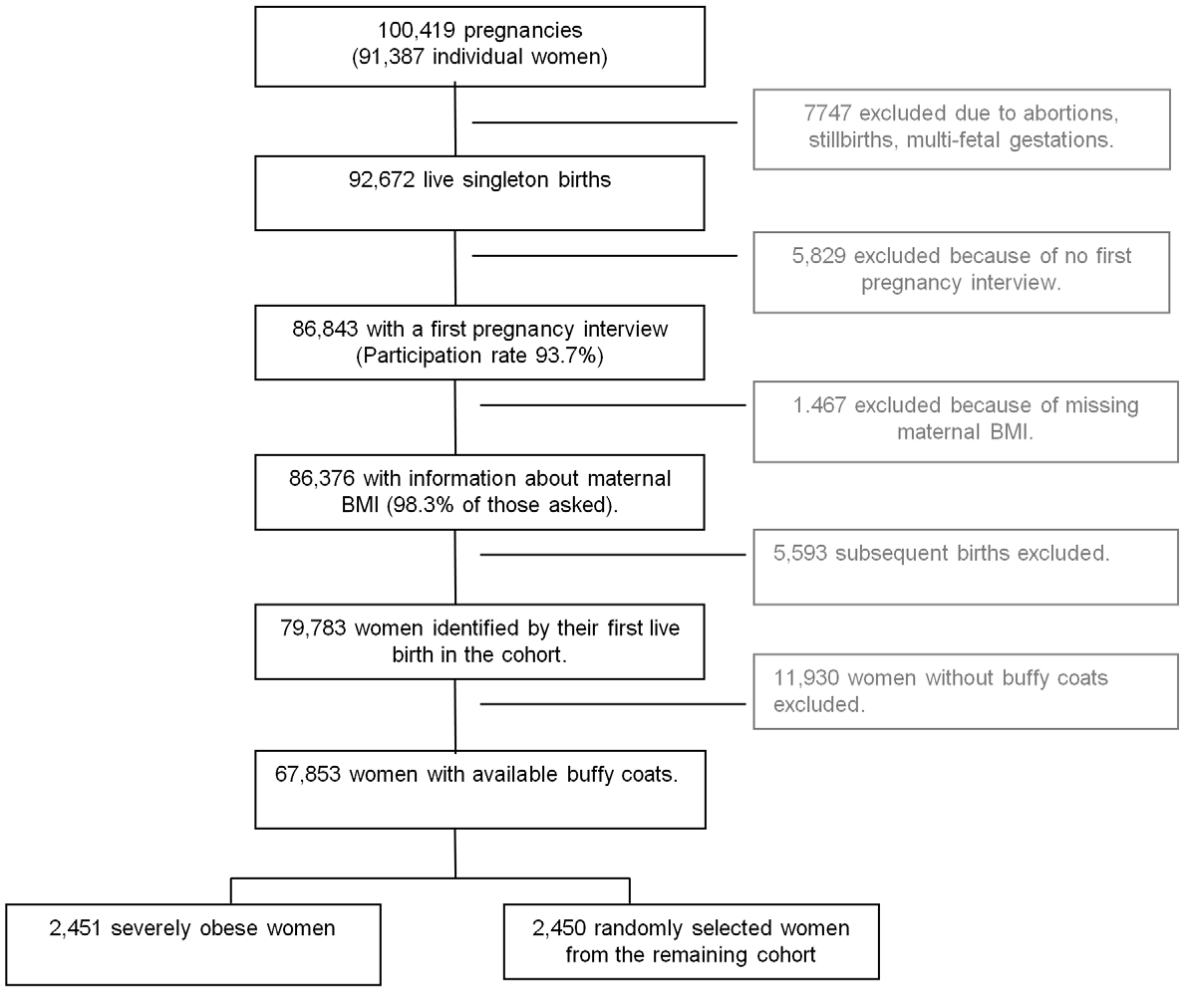
Study population sampled within the Danish National Birth Cohort 1996–2002.

The main exposure in the present study was prepregnant BMI based on self-reported information on prepregnancy weight and height from the first pregnancy interview. A flow diagram of the study population is presented in Figure 1. For women in the DNBC to be included in the present study, we initially requested that they had given birth to a liveborn singleton infant, that they had participated in the first pregnancy interview and that information about prepregnancy BMI were available (n = 79,783). We also requested that buffy coats were available for future genetic analyses, which left 67,853 women in the sampling frame. Within this sampling frame, we identified the 4% with the largest residuals from the regression of BMI on age and parity (all entered as continuous variables), and the BMI ranged among these women from 32.6 through 64.4. From the remaining cohort, we selected a random sample of similar size. Thus, the study population consisted of 2451 severely obese women (mean BMI 36.9) and 2450 randomly selected women (mean BMI 23.1) who functioned as a reference group for comparison with the obese group.

### Exposure Variables

In addition to a comparison between the obese group and the reference group, we also examined different degrees of obesity. To optimize the discriminative power, we divided the obese group into the following three categories of approximately equal size: (32.6≤BMI<35), (35≤BMI<37.5), (BMI≥37.5). Thus, the threshold between the first and second category equaled the threshold between obesity Class 1 and 2 (obesity and extreme obesity) according to the definitions of The World Health Organization (WHO) [13].

From the first pregnancy interview, we also used information about the woman's age at conception; parity; lifestyle habits in the first part of pregnancy including smoking, alcohol intake and physical exercise; and social status defined by education and occupation. From the interview 6 months after birth (participation rate 78.9%), we obtained information about her total gestational weight gain. The interview 18 months after birth (participation rate 73.3%) gave information about her partner's weight and height to calculate paternal BMI (available for 95% of those asked). The categorization of these variables is displayed in Table 1.

**Table 1:** Maternal characteristics according to severity of obesity.

	Reference women	Obese women		Obesity categories			
	BMI 15.0–33.3	BMI 32.6–64.4	*P* * *^′^*	BMI 32.6–35	BMI 35–37.5	BMI≥37.5	*P* * *^′^*
**Total population**	2450	2451		888	754	809	
**Prepregnancy weight**							
Mean (sd) in kg	66 (10.4)	104 (12.5)	*<0.01*	96 (7.3)	102 (7.8)	116 (12.6)	*<0.01*
**Age at conception**	%	%		%	%	%	
<25	12.7	16.1		15.2	17.5	15.8	
25–29	41.6	41.6		43.1	41.8	39.8	
30–34	34.9	32.0		30.6	30.4	35.1	
35+	10.8	10.2	*0.02*	11.0	10.3	9.3	*0.03*
**Parity**							
Primiparous	50.1	47.5		52.5	43.2	45.9	
Multiparous	49.9	52.6	*0.03*	47.5	56.8	54.1	*0.03*
**Height**							
<1.60 m	4.7	6.9		6.6	7.0	7.1	
1.60–1.69 m	47.7	51.9		49.4	53.6	53.2	
1.70 m+	47.6	41.2	*0.03*	43.9	39.4	39.8	*0.03*
**Gestational weight gain**							
<11kg	17.0	59.2		52.4	59.3	66.4	
11–19 kg	59.2	30.8		35.8	31.5	24.9	
>19 kg	23.8	10.0	*0.02*	11.9	9.2	8.8	*0.04*
**Smoking in pregnancy**							
Non smoker	84.0	83.1		83.9	82.0	83.3	
0–10 cig/day	12.7	11.1		11.4	12.2	9.7	
>10 cig/day	3.3	5.8	*0.04*	4.7	5.8	7.1	*0.04*
**Alcohol consumption**							
0 shots/wk	53.6	69.5		68.3	70.3	69.9	
1/2–3 shots/wk	43.8	29.7		30.3	29.1	29.7	
>3 shots/wk	2.7	0.8	*0.04*	1.4	0.7	0.4	*0.04*
**Exercise in pregnancy**							
No exercise	62.3	67.6		65.6	68.8	68.8	
1–180 min/wk	30.3	27.0		28.8	26.3	25.7	
180 min+/wk	7.4	5.4	*0.03*	5.6	4.9	5.6	*0.03*
**Social group**							
High	54.9	33.0		35.2	33.5	30.2	
Middle	36.0	48.7		49.5	47.5	48.9	
Low	9.1	18.3	*0.02*	15.4	19.0	20.9	*0.03*
**Partners BMI**							
<18.5	0.5	0.9		1.0	1.1	0.6	
18.5–24.9	53.5	36.9		39.7	37.3	33.5	
25.0–29.9	39.7	43.1		42.4	42.5	44.4	
30+	6.3	19.1	*0.03*	17.0	19.1	21.6	*0.03*

* Tests for no association were for continuous variables T-test and ANOVA (analysis of variance), for categorical variables test for trend based on Goodman and Kruskal's gamma statistics. In tests applied to the 3 obesity groups, reference women were excluded.

Subjects with missing values: exercise, n = 38; gestational weight gain, n = 1781; paternal BMI, n = 1498. Missing for other variables, n<10.

### Maternal Outcomes

From the first pregnancy interview, we obtained information about use of infertility treatment prior to this pregnancy and for planned and partly planned pregnancies also about waiting time to pregnancy. A waiting time to pregnancy >1 year was used as a measure of subfecundity [14]. From the second pregnancy interview (participation rate 93.6%), we used self-reported information about urinary tract infections during pregnancy.

By linkage to the National Hospital Discharge Register (NHDR) and use of codes from the International Classification of Diseases, 10^th^ Revision (ICD-10), we identified pregnancies complicated by preeclampsia or eclampsia (O14 or O15), pregnancies with chronic or gestational hypertension (I10 through I15 and O10, O11, and O13), and pregnancies with a diagnosis of gestational diabetes (O24). For the latter disease, we expected some underreporting in the NHDR and added self-reported information from the pregnancy interviews. Gestational age as recorded in the NHDR at birth was used to classify timing of birth, which was divided into ‘preterm birth’ (<37 full weeks), ‘birth at term’ (37–41 full weeks), and ‘postterm birth’ (>41 full weeks). Birth complications were also identified in the NHDR. They included instrumental deliveries, which in nearly all cases covered vacuum extraction, and caesarean deliveries, which were divided according to whether they were carried out before labor (planned) or during labor (emergency).

Based on the women's report on duration of breastfeeding in the interview 6 months after birth we generated the following outcomes: ‘Full breast feeding <2 weeks’ was used to measure inability to initiate breastfeeding. In those women who initiated breastfeeding, ‘full breastfeeding <14 weeks’ was used to measure inability to sustain breastfeeding when initiated. This threshold was used because Danish Health Authorities during the study period recommended women not to introduce complementary foods before 4–6 months of age, and Danish women are strongly supported to fully breastfeed until then. From the interview 6 months after birth, we also used information about the woman's weight to calculate postpartum weight retention as the difference between the woman's prepregnancy weight and the weight reported 6 months after birth. Postpartum weight retention was summarized by two variables defined as postpartum weight loss (loss ≥5 kg) and postpartum weight retention (gain of ≥5 kg) relative to a woman's prepregnancy weight. We also calculated postpartum weight retention at 18 months for those women in the study population who participated in the second postpartum interview, who had not given birth again and who were not pregnant again. Here we used the same cutoff points as for 6 months.

### Neonatal Outcomes

Neonatal outcomes were identified in the National Birth Registry and included birth weight, length, Apgar score after 5 minutes, and congenital anomalies (ICD-10 codes) diagnosed before one year of age. Birth weight was standardized for gestational age by calculating a z-score and dichotomized into either a small-for-gestational age infant (z-score<10^th^ percentile) or a large-for-gestational age infant (z-score>90^th^ percentile). To describe fatness of the infant, we calculated ponderal index of the newborn (birth weight in grams divided by the birth length in cm cubed) (30), which was dichotomized into either low ponderal index (values<10^th^ percentile) or high ponderal index (values>90^th^ percentile). Low Apgar score was defined as a value<8 after 5 min. From the interview 18 months after birth, we had information about infant weight and height at 12 months, which was used to calculate BMI. Infant obesity was defined as a BMI≥95^th^ percentile. We also estimated catch-up growth by subtracting birth weight from weight at one year of age. Values≥95^th^ percentile were classified as ‘high catch-up growth’.

### Statistical Methods

We used χ^2^ test for trend (Goodman and Kruskal's gamma statistics) to compare the distribution of maternal characteristics in obese women and reference women and the distributions across the three obesity groups.

Next, we used multinomial logistic regression models to estimate odds ratios (OR) for the association between obesity and the selected maternal and neonatal outcomes, either with all obese women as the exposed group or with obese women divided into three categories according to severity. In both models, the reference women were the comparison group. Two sets of analyses were carried out. In a first series, the models were only adjusted for age and parity, which were used as basis for selection of the obese group. In a second series of analysis, the models were in addition adjusted for a number of potential confounders including maternal height, smoking, alcohol consumption, physical exercise, and social group, and for infant obesity also for paternal BMI. This strategy was chosen because several of the variables added to the second series may also be regarded as mediators of the effect of obesity on reproductive health, and the adjustment may thereby diminish the effects of obesity. Because the two sets of analyses produced almost similar results, only the results from the fully adjusted model are presented here, but results from the first series are available on request. For postpartum weight retention, we included gestational weight gain in an additional analysis to be able to compare women with the same gain, although this may also regarded an overadjustment given the low gain in obese women. To test for a trend in the change of risk across the three obesity categories, we repeated this analysis after excluding the reference group and with the exposure variable entered as a continuous variable. We used a significance level of 0.05 in all statistical tests, and OR are presented with 95% confidence intervals (CI). We used STATA software (version 9.1 Special Edition; Stata Corp; College Station, TX) for all statistical analysis.

## Results

Compared to other women, obese women were slightly younger, shorter of height and of higher parity (Table 1). During pregnancy, they were more often heavy smokers, drank less alcohol, exercised less and had lower gestational weight gain. They were of lower social status, and more often they had a partner who was also overweight or obese. All these differences also correlated with the level of obesity.

Prior to pregnancy, obese women more often received infertility treatment than other women, and among women with planned pregnancies, obese women had a higher risk of waiting more than one year to become pregnant (Table 2). The adjusted odds ratios across obesity categories showed that the increased use of infertility treatment decreased slightly with increasing severity of obesity while increased risk of a long waiting time to pregnancy was rather stable across obesity categories.

**Table 2:** Adjusted odds ratios (OR) for selected pregnancy outcomes according to severity of maternal obesity.

	Reference women (n = 2450)	Obese women BMI 32.6–64 (n = 2451)					Obese category 1 BMI 32.6–35 (n = 888)					Obese category 2 BMI 35–37.5 (n = 754)					Obese category 3 BMI≥37.5 (n = 809)					
	Crude risk	Crude risk	Adj. OR*	95% CI			Crude risk	Adj. OR*	95% CI			Crude risk	Adj. OR*	95% CI			Crude risk	Adj. OR*	95% CI			*P* †
**Pregnancy-related disease**																						
Time to pregnancy>1y§	*13.8*	*26.6*	**2.2**	1.8	;	2.6	*28.3*	**2.3**	1.9	;	2.8	*24.7*	**2.1**	1.7	;	2.6	*26.5*	**2.1**	1.7	;	2.7	*0.55*
Infertility treatment	*5.7*	*9.3*	**1.7**	1.4	;	2.1	*11.0*	**1.9**	1.4	;	2.6	*9.3*	**1.8**	1.3	;	2.5	*7.5*	**1.3**	1.0	;	1.9	*0.06*
Urinary tract infections$	*11.7*	*14.8*	**1.3**	1.1		1.5	*13.6*	**1.2**	0.9		1.5	*14.8*	**1.3**	1.0		1.6	*16.2*	**1.4**	1.1	;	1.8	*0.24*
Type 2 diabetes	*1.39*	*6.4*	**4.6**	3.1	;	6.7	*5.9*	**4.2**	2.7	;	6.6	*6.4*	**4.7**	3.0	;	7.5	*6.9*	**4.8**	3.1	;	7.5	*0.57*
Preeclampsia	*2.0*	*5.6*	**2.9**	2.0	;	4.1	*5.1*	**2.5**	1.6	;	3.8	*4.4*	**2.3**	1.4	;	3.6	*7.3*	**3.9**	2.6	;	5.9	*0.02*
Other hypertension	*1.2*	*3.8*	**3.3**	2.1	;	5.1	*2.0*	**1.7**	0.9	;	3.1	*4.4*	**4.0**	2.4	;	6.7	*5.2*	**4.7**	2.8	;	7.7	*<0.01*
**Timing of birth (full weeks)**																						
Preterm (<37 )	*3.9*	*4.4*	**1.2**	0.9	;	1.6	*3.9*	**1.0**	0.6	;	1.5	*4.4*	**1.3**	0.9	;	2.0	*4.8*	**1.3**	0.9	;	1.9	*0.20*
Term (37–41)	*68.0*	*60.9*	**1.0**	ref			*63.2*	**1.0**	ref			*58.9*	**1.0**	ref			***60.2***	**1.0**	ref			
Postterm (>41)	*28.1*	*34.8*	**1.4**	1.2	;	1.6	*32.9*	**1.3**	1.1	;	1.5	*36.7*	**1.5**	1.3	;	1.8	*35.0*	**1.4**	1.2	;	1.7	*0.25*
**Birth complications**																						
Spontaneous delivery	76.0	*65.4*	**1.0**	ref			*####*	**1.0**	ref			*66.2*	**1.0**	ref			***63.7***	**1.0**	ref			
Planned caesarean	7.5	*10.5*	**1.6**	1.3	;	2.0	*####*	**1.5**	1.1	;	2.0	*10.1*	**1.6**	1.2	;	2.2	*11.3*	**1.8**	1.4	;	2.4	*0.19*
Emergency cesarean	7.9	*14.3*	**2.1**	1.8	;	2.6	*####*	**1.9**	1.5	;	2.5	*15.0*	**2.4**	1.8	;	3.1	*14.1*	**2.2**	1.7	;	2.9	*0.36*
Instrumental delivery	8.7	*9.8*	**1.3**	1.1	;	1.7	*9.5*	**1.2**	0.9	;	1.6	*8.8*	**1.3**	1.0	;	1.8	*11.0*	**1.6**	1.2	;	2.1	*0.10*
**6 months after birth** ‡																						
Breastfeeding																						
Failure to initiate (<2w)	*14.9*	*29.3*	**2.2**	1.8	;	2.6	*26.6*	**1.9**	1.5	;	2.4	*28.9*	**2.1**	1.7	;	2.7	*32.6*	**2.6**	2.1	;	3.2	*0.01*
Failure to sustain (2–14w)	*15.3*	*29.8*	**2.2**	1.8	;	2.6	*30.0*	**2.2**	1.7	;	2.8	*25.4*	**1.8**	1.3	;	2.3	*33.9*	**2.6**	2.1	;	3.4	*0.18*
Weight retention ≥5 kg	*21.2*	*14.6*	**0.6**	0.5	;	0.7	*15.4*	**0.6**	0.5	;	0.7	*16.6*	**0.6**	0.5	;	0.8	*11.8*	**0.4**	0.3	;	0.6	*0.49*
Weight loss ≥5 kg	*6.0*	*38.4*	**9.8**	7.9	;	12.3	*32.2*	**7.5**	5.8	;	9.8	*36.7*	**9.1**	6.9	;	11.9	*47.2*	**14.4**	11.0	;	18.7	*<0.01*
18 months after birth**																						
Weight retention ≥5 kg	*12.8*	*13.2*	**1.0**	0.8	;	1.2	*13.9*	**1.0**	0.8	;	1.3	*11.9*	**0.9**	0.6	;	1.2	*13.8*	**1.0**	0.7	;	1.4	*0.22*
Weight loss ≥5 kg	*10.7*	*46.6*	**7.7**	6.3	;	9.4	*41.5*	**6.3**	5.0	;	8.1	*45.8*	**7.3**	5.7	;	9.4	*53.4*	**10.5**	8.1	;	13.5	*<0.01*

* Compared to reference women. Adjusted for age, parity, height, smoking, alcohol consumption, physical exercise, social group, and for postpartum retention also time of interview in weeks after birth.

† Test for trend, only included adjusted odds ratios in the 3 obesity groups.

§ Only planned pregnancies (n = 4278).

$ Only available in women with a 2nd pregnancy interview (n = 4564).

‡ Only available in women with an interview 6 m after birth, (n = 3627 for weight retention/loss, n = 3865 for failure to initiate breastfeeding, n = 3000 for failure to continue (only women who initiated)).

During pregnancy, obese women had a higher risk of urinary tract infections, gestational diabetes, preeclampsia and other hypertensive disorders, and the excess risk increased with increasing degree of obesity – especially for preeclampsia and other hypertensive disorders (p-values (test for trend) 0.02 and <0.01, respectively).

Obese women had more often a prolonged pregnancy (>41 full weeks), and tended also to have a slightly increased risk of preterm birth. An elevated risk of birth complications such as cesarean delivery, especially on an emergency basis, and instrumental deliveries was observed in obese women, again with an increasing occurrence with higher obesity categories.

After birth, obesity was associated with failure to initiate breastfeeding with the risk increasing with the severity of obesity (p (test for trend) 0.01). Among women that initiated breastfeeding, failure to sustain breastfeeding beyond 14 weeks was far more frequent in obese women, especially in those in the highest obesity category (Adj. OR 2.6; 95% CI: 2.1–3.4).

Half a year after birth, the crude risk of retaining 5 kg or more relative to one's prepregnancy weight was considerably lower in obese women than in other women (14.6% vs 21.2%). However, after adjustment for gestational weight gain, which was considerably lower in obese women, the risk in obese women equaled the risk in other women (Adj. OR 1.0; 95% CI: 0.8–1.3). Notably, the crude risk of weight retention 18 months after birth was similar in obese women and in the reference group (13.2% vs. 12.8%), but after adjustment for gestational weight gain, risk in obese women tended to be higher than in other women, especially in women in the lowest obesity category (Adj. OR 1.4; 95% CI: 1.0–2.0). Both 6 and 18 months after birth, chance of having lost 5 kg or more relative to one's prepregnancy weight was very high in obese women and increased with severity of obesity. Although chance of having lost 10 kg or more half a year after birth was also much higher in obese women than in other women (19.0% vs. 1.1%), only 4.1% of women initially defined as obese in this study had a BMI<30 such that they actually had changed BMI category and were no longer obese.

The risk of having a small baby was lower in obese women while the risk of having a large baby was increased (Table 3). Measures based on ponderal index showed the same pattern with fewer babies with low values and more babies with high values in obese women. For LGA and high ponderal index, the highest risks were seen in women in the highest obesity category (BMI≥37.5). Infants of obese women also had a higher risk of low Apgar score 5 minutes after birth (Adj. OR 1.6; 95% CI 1.0–2.7), which however was highest in the lowest obesity category (Adj. OR 2.0; 95% CI 1.1–3.6), but also estimated with large imprecision because of the low incidence of the outcome in the study population. The risk of congenital anomalies, when measured as all anomalies combined in one group, was similar in obese women and other women and across obesity categories.

**Table 3:** Adjusted odds ratios (OR) for neonatal and infant outcomes according to severity of maternal obesity.

	Reference women (n = 2450)	Obese women BMI 32.6–64 (n = 2451)					Obese category 1 BMI 32.6–35 (n = 888)					Obese category 2 BMI 35–37.5 (n = 754)					Obese category 3 BMI≥37.5 (n = 809)					
	Crude risk	Crude risk	Adj. OR*	95% CI			Crude risk	Adj. OR*	95% CI			Crude risk	Adj. OR*	95% CI			Crude risk	Adj. OR*	95% CI			*P* †
**Neonatal outcomes**																						
SGA (<10 perentile)	*10.9*	*7.6*	**0.6**	0.5	;	0.8	*8.0*	**0.7**	0.5	;	0.9	*7.2*	**0.6**	0.4	;	0.8	*7.5*	**0.6**	0.5	;	0.8	*0.79*
LGA (>90 percentile)	*11.8*	*21.5*	**2.3**	1.9	;	2.7	*19.7*	**2.1**	1.7	;	2.6	*20.0*	**2.1**	1.7	;	2.6	*24.7*	**2.8**	2.2	;	3.4	*0.01*
Low ponderal index	*9.9*	*8.1*	**0.7**	0.6	;	0.9	*9.3*	**0.9**	0.7	;	1.1	*8.0*	**0.7**	2.7	;	1.0	*6.7*	**0.6**	0.4	;	0.8	*0.07*
High ponderal index	*9.8*	*15.6*	**1.7**	1.4	;	2.1	*14.7*	**1.7**	1.3	;	2.1	*14.8*	**1.6**	1.2	;	2.1	*17.4*	**2.0**	1.6	;	2.5	*0.25*
Low Apgar Score	*1.2*	*1.9*	**1.6**	1.0	;	2.7	*2.4*	**2.0**	1.1	;	3.6	*1.6*	**1.5**	0.7	;	3.0	*1.5*	**1.3**	0.6	;	2.6	*0.23*
Congenital anomalies	*5.6*	*5.8*	**1.0**	0.8	;	1.3	*5.3*	**0.9**	0.6	;	1.3	*6.4*	**1.1**	0.8	;	1.6	*5.8*	**1.0**	0.7	;	1.4	*0.68*
**Infant outcomes at one year of age** §																						
Infant obesity$	*4.8*	*8.2*	**1.9**	1.3	;	2.6	*7.7*	**1.7**	1.1	;	2.7	*7.4*	**1.6**	1.0	;	2.6	*9.6*	**2.2**	1.5	;	3.4	*0.24*
High catch-up growth‡	*3.5*	*5.0*	**1.4**	1.0	;	2.1	*4.5*	**1.3**	0.8	;	2.2	*5.8*	**1.8**	1.1	;	3.0	*4.6*	**1.3**	0.8	;	2.3	*0.99*

* Compared to reference women. Adjusted for age, parity, height, smoking, alcohol consumption, physical exercise, social group and for infant obsity also parental BMI.

† Test for trend, only included adjusted odds ratios in the 3 obesity groups.

§ Only available in women with an interview 18 m after birth, (n = 2892 for infant obesity, n = 2760 for high catch-up growth).

$ Infant obesity defined as BMI>95. percentile.

‡ High catch-up growth defined as growth>95. percentile (weight at one year - birthweight).

Abbreviations: SGA, small-for-gestational-age; LGA, large-for-gestational-age.

One year after birth, infants of obese mothers had a higher risk of obesity themselves measured as a BMI≥95 percentile (Adj. OR 1.9; 95% CI: 1.3–2.6), especially in mothers with BMI≥37.5 (Adj. OR 2.2; 95% CI: 1.5–3.4). Also high catch-up growth was increased in infants of obese mothers, but with no trend across obesity categories.

## Discussion

In this study, we report excess risks of a wide range of important maternal, neonatal and infant outcomes in severely obese women. For most of these outcomes, the risk increased with increasing degree of obesity. It is, however, unknown what the excess risk would have been had the women not been obese. The etiology behind the wide range of reproductive outcomes related to maternal obesity is still poorly understood. Obesity is associated with a range of metabolic, inflammatory and vascular abnormalities that may disturb the development of a normal pregnancy and perhaps even increase the susceptibility of the fetus for later disease. On the other hand, it is well known that obesity has a considerable genetic component, and these genetic traits or other causes of obesity may influence reproductive outcomes as well as obesity. Finally, social characteristic of obese individuals as well as their diet- and exercise habits typically differ from those of non-obese individuals. Although we adjusted for a number of important confounders that have not been available in previous studies, we expect some uncontrolled confounding to remain.

The large variety of data provided by the DNBC allowed us to study more outcomes within the same population than was done previously. The study adds new information about the relationship between different degrees of severe obesity and a number of outcomes that only have been sparsely studied. Most previous studies on severe obesity have been small or based on secondary data, which has restricted the ability to study outcomes not routinely measured during prenatal care.

Our study has, however, also some limitations. Due to the sampling strategy and the size of the obese group, we were only partly able to adhere to the WHO obesity categories [13]. However, even WHO's categories are arbitrary thresholds, inserted as round numbers in a continuum. As the scientific objectives of the present study were to elucidate the association in the extreme tail of the BMI distribution, we chose a strategy that allowed us to do so with the highest discriminative power. Furthermore, we relied. on self-reported information about prepregnancy BMI. We previously validated prepregnancy weight relative to the weight observed in antenatal care and found a small but consistent underreporting on the entire BMI-scale of an average of 0.66 kg ranging from 0.44 for a prepregnancy weight of 50 kg and 0.96 kg for a prepregnancy weight of 100 kg [15]. However, BMI categories derived from the two BMI estimates agreed in 91% of cases. Some of the measured outcomes were also self-reported such as breastfeeding and infant weight and risk of information bias should be considered. However, we believe that misclassification would most likely be non-differential and - if differential – obese women would probably tend to underreport the weight of their child to a larger extent than other women. In both cases, bias of the association would be towards the null.

We do not think that the study is seriously affected by selection problems since the women chose to take part in the DNBC early in pregnancy when the outcomes under study were not known. Furthermore, obese women were only slightly underrepresented compared to the general female population aged 25–44 years (8.4% vs. 9.1%) [16]. To reach the final study population, we included only pregnancies ending in liveborn singletons. It has been shown that obesity is associated with miscarriages and stillbirths [17] and also with multiple pregnancies [18], which we were not able to address. Also, the study population was restricted to women with available blood samples, but excluded women were equally distributed in the groups we compared. Finally, our reference group was sampled randomly from all in the remaining cohort and included some women who were also obese (BMI≥30, n = 115). However, all results were almost similar to those reported when these women were excluded.

We found, as have others [2], [19], [20], that obese women had longer waiting time to pregnancy and used infertility treatment more frequently [21], but we saw no dose response pattern with increasing obesity. A Dutch study of pregnancy occurrence in 6000 subfertile couples found, however, that the ability to conceive spontaneously was increasingly impaired across the entire range of obesity [20], which may illustrate the limitations in using a pregnancy sample.

The strong positive association between increasing obesity and risk of pregnancy diseases with a vascular pathology such as preeclampsia and other hypertensive disorders are in keeping with what others have reported [21]–[25], as was the strong association between severe obesity and gestational diabetes [21], [26]. We also identified an increased risk of urinary tract infections during pregnancy across obesity categories. This outcome has only been addressed in two publications [4], [27] and not in such detail, but their findings support these observations. Obese women have been found to be more susceptible to infections, which may be due to a reduced immune function [28]. The increased risk of postdatism in severely obese women has been observed by others [22], [23] as has an increased risk of preterm birth [21]–[23], which we, however, could not identify. Previous studies also identified an elevated risk of birth complications with an increasing tendency in severely obese women [21]–[23], [25].

As others have shown [5], [29]–[31], we found obesity to be strongly associated with both failure to initiate and sustain full breastfeeding, especially in the heaviest women. It has been suggested that obesity impairs development of the mammary glands both before and during pregnancy, but also endocrine, medical and psychosocial factors may play a role [32].

We found that severely obese women were at decreased risk of weight retention 6 months after birth. However, when they were compared with other women with similar gestational weight gains, their risks were equal, and 18 months after birth, their risk tended to be higher, especially in obese women within the lowest category. This is in accord with findings by others [6], [33] and may indicate that even among severely obese women, some are still on a steeper growth trajectory than other women. A 15 year follow-up study in Sweden showed, however, that overweight women had no excess risk of postpartum weight retention [34].

Our findings of decreasing risk of being born with a low birth weight and an increasing risk of being born with a high birth weight with increasing degree of severe obesity are in accord with several other studies [21]–[23]. We also found the same pattern for low and high ponderal index which indicates that the observed increase in birth weight across maternal obesity categories is associated with fatness of the baby.

To define infant obesity is controversial, and there is no consensus on how to measure it [35], but maternal obesity appears to be associated with infant obesity [36], [37] and also high catch-up growth [38]. The observed associations are of concern because it may indicate a higher risk of childhood obesity [35], [39], [40] and a cardiovascular and metabolic risk profile in childhood or early adulthood [41]–[43].

Overall, we present data from a newly available and well characterized cohort on severe obesity as a potential causal risk factor for a large collection of reproductive outcomes. If some of the presented associations are causal, the health consequences of the obesity epidemic are scaring. The missing indication for such a concurrent epidemic of these reproductive failures calls, however, for caution in expecting that these adverse outcomes can be eliminated by inducing weight loss. Still, the associations may be useful in clinical practice for making predictions at the individual level. Future studies should include information on functional biological pathways and gene variants associated with severe obesity.
